# Draft Genome Sequences of Nine Clinical Isolates of Vancomycin-Resistant Enterococci

**DOI:** 10.1128/genomeA.00803-16

**Published:** 2016-08-18

**Authors:** Paul G. Higgins, Daniela Koehler, Jacqueline Z. M. Chan, Oliver A. Cornely, Gerd Fätkenheuer, Meyke Gillis, Mark J. Pallen, Johanna Tien, Harald Seifert, Maria J. G. T. Vehreschild, Andrew D. Millard

**Affiliations:** a Institute for Medical Microbiology, Immunology and Hygiene, University of Cologne, Cologne, Germany; b German Center for Infection Research (DZIF), Partner Site Bonn-Cologne, Cologne, Germany; c Department of Anesthesiology and Intensive Care Medicine, University Hospital of Cologne, Cologne, Germany; d Microbiology and Infection Unit, Warwick Medical School, University of Warwick, Warwick, United Kingdom; e Department I for Internal Medicine, University Hospital of Cologne, Cologne, Germany; f Cologne Excellence Cluster on Cellular Stress Responses in Aging-Associated Diseases (CECAD), and Clinical Trials Centre Cologne (ZKS Köln), University of Cologne, Cologne, Germany

## Abstract

In 2012, there was an increase in vancomycin-resistant enterococci (VRE) isolated from the intensive care unit at the University Hospital of Cologne. Using whole-genome sequencing it was possible to establish that bloodstream infections with VRE were not the result of an outbreak or cross infections.

## GENOME ANNOUNCEMENT

Bloodstream infections caused by vancomycin-resistant enterococci (VRE) are a growing problem in specialized hospital units and can result in a high mortality rate (1). Starting in late 2012 an increasing number of patients were colonized or infected with VRE in the intensive care unit at the University Hospital of Cologne. Nine isolates were collected from eight patients. Preliminary pulse field gel electrophoresis analysis assigned eight isolates to two distinct pulsotypes, A and B, suggesting clonal transmission as the source for the increased cases. Initial multilocus sequence typing (MLST) analysis indicated that all isolates belong to the hospital-adapted clonal complex (CC) 17 (2). Whole-genome sequencing was carried out to further investigate these strains.

Bacterial genomic DNA was prepared from cultures using the QIAGEN DNeasy kit according to the manufacturer’s instructions, and sequenced using MiSeq (Illumina, USA). One nanogram of genomic DNA was prepared using the Nextera XT DNA sample preparation kit (Illumina) prior to sequencing on the MiSeq platform using the paired-end 2 × 250-bp protocol. The resulting FASTQ files were assembled with SPAdes version 3.7 with the following option “-careful” (3). Postassembly filtering removed all contigs less than 500 bp with less than 5× coverage. All genomes were sequenced to a minimum depth of 24×. Resultant contigs were annotated with Prokka version 1.11 using the *Enterococcus*-specific database for annotation (4). Core-genome analysis was carried out with REALPHY (5) with the following settings “-quality 25, -genes, -ref IS18,” with all other settings left as default. Putative antimicrobial resistance genes were predicted using the CARD database (6). *In silico* MLST from the genomic data confirmed that the isolates belong to CC17 with ST17, ST203, and ST117 represented. Furthermore, genome sequencing confirmed that all isolates carried vancomycin resistance genes, either VanA (IS18, IS19, IS20, IS21, and IS30) or VanB (IS12, IS17, IS23, and IS25) genotype, with genes conferring resistance to gentamicin, streptomycin, and erythromycin found in all isolates. Additionally, isolate IS19 carried the *dfrA* gene, suggesting resistance to trimethoprim. Utilizing REALPHY, 2,038,337 sites were identified that were shared between all nine isolates; these were further filtered to include only intragenic sites. The number of polymorphisms detected between strains varied from 34 (between IS21 and IS30) and 7,669 (IS19 and IS12). The large number of polymorphisms combined with variable carriage of antibiotic resistance genes suggests that this was not a clonal outbreak of VRE.

### Accession number(s).

The draft genome sequences have been deposited in DDBJ/ ENA/GenBank under the accession numbers FKLH01000001 (IS12), FKLC01000001 (IS17), FKLG01000001 (IS18), FKLF01000001 (IS19), FKLJ01000001 (IS20), FKLK01000001 (IS21), FKLE01000001 (IS23), FKLD01000001 (IS25), and FKLI01000001 (IS30).
